# Is There an Added Neonatal Risk in Vacuum-Assisted Deliveries with Nuchal Cord?

**DOI:** 10.3390/jcm11236970

**Published:** 2022-11-25

**Authors:** Hanoch Schreiber, Gal Cohen, Nir Mevorach, Maya Shavit, Michal Kovo, Tal Biron-Shental, Ofer Markovitch

**Affiliations:** 1 Department of Obstetrics and Gynecology, Meir Medical Center, 59 Tchernichovsky St., Kfar Saba 44281, Israel; 2 Sackler School of Medicine, Tel Aviv University, Tel Aviv 69978, Israel

**Keywords:** vacuum-assisted delivery, operative delivery, nuchal cord, cord pH

## Abstract

This retrospective cohort study assessed the association between nuchal cord and adverse outcomes during vacuum-assisted delivery (VAD). Women with singleton pregnancies, 34–41-weeks gestation, who underwent VAD, from 2014 to 2020 were included. The primary outcome was umbilical cord pH ≤ 7.1. Secondary outcomes were neonatal intensive care unit admission, Apgar scores, pH < 7.15, subgaleal hematoma, shoulder dystocia and third/fourth-degree perineal tear. Outcomes were compared between neonates with (1059/3754, 28.2%) or without (71.8%) nuchal cord after VAD. No difference in cord pH ≤ 7.1 was found between groups. The nuchal cord group had a lower rate of nulliparity (729 (68.8%) vs. 2004 (74.4%), *p* = 0.001) and higher maternal BMI (23.6 ± 4.3 vs. 23.1 ± 5, *p* = 0.017). Nuchal cord was associated with higher rates of induction (207 (19.5%) vs. 431 (16%), *p* = 0.009) and lower birthweights (3185 ± 413 vs. 3223 ± 436 g, *p* = 0.013). The main indication for VAD in 830 (80.7%) of the nuchal cord group was non-reassuring fetal heart rate (NRFHR) vs. 1989 (75.6%) controls (*p* = 0.004). The second stage was shorter in the nuchal cord group (128 ± 81 vs. 141 ± 80 min, *p* < 0.001). Multivariate regression found nulliparity, induction and birthweight as independent risk factors for nuchal cord VAD. Although induction and NRFHR rates were higher in VAD with nuchal cord, the rate of umbilical cord acidemia was not.

## 1. Introduction

Nuchal cord is a common finding at delivery, with an incidence of approximately 20% of all term singleton deliveries [1,2]. Usually, it is not associated with added risk of adverse outcomes [3,4].

In view of the progress in ultrasonography, nuchal cord may be diagnosed prenatally with good sensitivity and specificity [5,6]. Yet, according to the guidelines of the American Institute of Ultrasound in Medicine and the International Society of Ultrasound in Obstetrics and Gynecology, scanning the course of the umbilical cord is not part of the routine prenatal scan, as it can be considered a variant of the norm [7,8]. Moreover, prenatal screening for nuchal cord might lead to maternal anxiety and unnecessary appointments for fetal assessment and sonographic follow-ups. It also may cause interventions in the absence of evidence of increased risk for fetal adverse outcomes or of the benefit of interventions [9].

Nonetheless, possible sequalae for nuchal cord include higher frequency of non-reassuring fetal heart rate (NRFHR) and increased rate of operative deliveries compared to pregnancies without nuchal cord [10,11]. Multiple loops and tight nuchal cord are associated with adverse neonatal outcomes [12,13,14], although possible biases might be related to changes in cord “tightness” during the labor process and uneven documentation, performed mostly when fetal asphyxia is suspected.

Our hypothesis was that nuchal cord may be associated with increased adverse neonatal outcomes during vacuum-assisted delivery (VAD). In the presence of nuchal cord, the rapid pulling action during VAD may lead to cord compression, increased tightness, or even detrimental effects on its integrity. This study evaluated whether the presence of nuchal cord is associated with increased adverse neonatal outcomes when VAD is performed.

## 2. Materials and Methods

This retrospective cohort study included all singleton VAD that took place in one institution from 2014 to 2020. Exclusion criteria were VAD before 34 weeks, cesarean section for any reason (including failed vacuum) and antepartum fetal demise.

### 2.1. Data

Maternal characteristics, demographics and associated pregnancy complications, as well as delivery outcomes, were retrieved from the electronic medical records. Immediately after VAD, all neonates were examined by a pediatrician. Birthweight percentile for gestational age was assigned using local growth charts [15]. Small for gestational age (SGA) was defined as birthweight < 10th percentile for gestational age. The following information was collected from the neonatal records: Apgar scores, cord blood pH, neonatal intensive care unit (NICU) admissions, pH < 7.15, and subgaleal hematoma.

### 2.2. Vacuum-Assisted Delivery (VAD)

VAD was indicated to expedite vaginal delivery in cases of NRFHR or in cases of prolonged second stage or maternal exhaustion. Either a Kiwi Omnicup hand-pump or a Silastic cup were used during the entire study period. According to departmental protocol, before a VAD, the attending obstetrician performs a full evaluation of maternal and fetal parameters, including adequacy of the maternal pelvis, estimated fetal weight, fetal head position and station by vaginal examination and ultrasound to determine the appropriateness of VAD in each case. Note that for historic reasons, fetal head station was defined by thirds from −3 to +3. However, vacuum delivery, in accordance with ACOG recommendations, was performed only when the head station was 2 cm or lower. Each VAD was fully documented at the end of the procedure. In addition, the course of the umbilical cord was examined routinely in every delivery. Nuchal cord and true knots were documented in the medical record.

Neonatal outcomes were compared between neonates born with (nuchal cord group) or without (control group) nuchal cord during VAD.

### 2.3. Statistical Analysis

Nominal data are described as number and percentage. Continuous parameters are described by means and standard deviations. Metric variables were analyzed with *t*-test. Discrete variables were analyzed using the chi-squared test. *p* < 0.05 was considered statistically significant. Statistical analyses were performed with SPSS-25 software (IBM Corp., Armonk, NY, USA).

### 2.4. Power Analysis

Cord pH ≤ 7.1 was chosen as the primary outcome. The incidence of umbilical cord pH ≤ 7.1 was 4% in the study population. We decided that a two-fold increase in the rate of umbilical cord pH ≤ 7.1 from 4% to 8% would be considered a significant difference between those with or without nuchal cord. Therefore, a sample of 552 women in each group would be sufficient to detect a significant difference in the rate of umbilical cord pH ≤ 7.1 with a type I error of 5% and at least 80% power.

## 3. Results

**Figure 1:**
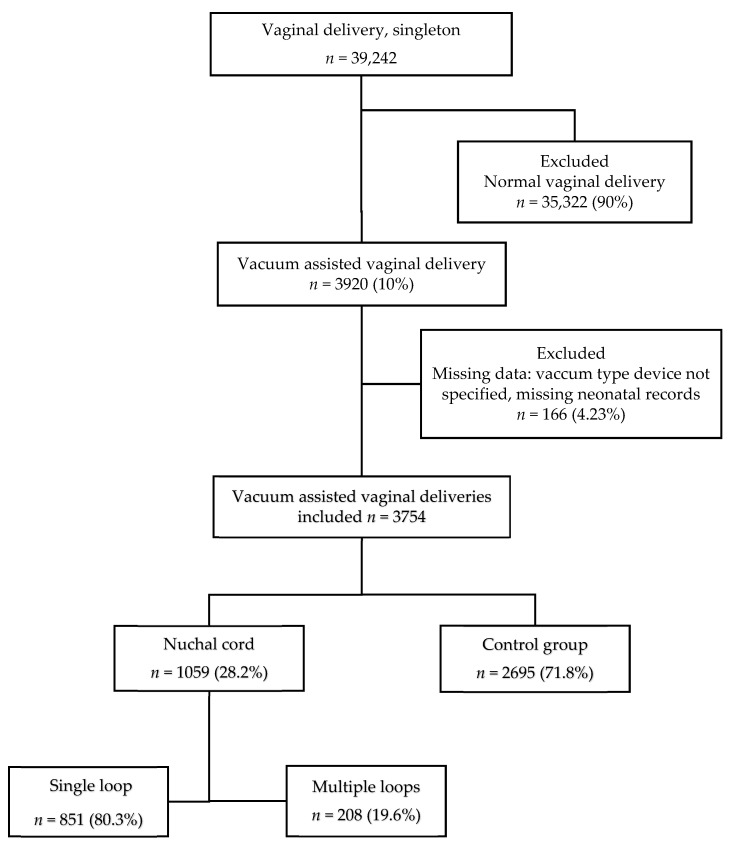
Flow diagram of study participants.

During the study period, 3920 VAD in singleton pregnancies were performed in our institution. Among them, 3754 met the inclusion criteria and were included in the study. Nuchal cord was documented in 1059 deliveries (Figure 1).

Compared to the control group, the nuchal cord group was characterized by a lower rate of nulliparity (729 (68.8%) vs. 2004 (74.4%), *p* = 0.001) and by higher maternal BMI (kg/m^2^) (23.6 ± 4.3 vs. 23.1 ± 5, *p* = 0.017). The other demographic characteristics were similar between the two groups (Table 1).

Compared to controls, the nuchal cord group was associated with higher rates of induction of labor (207 (19.5%) vs. 431 (16%), respectively; *p* = 0.009) and lower mean birthweight (3185 ± 413 g vs. 3223 ± 436 g, respectively; *p* = 0.013).

The main indication for VAD in 830 (80.7%) cases within the nuchal cord group was NRFHR, compared to 1989 (75.6%) in the control group (*p* = 0.004). Second-stage duration was also shorter in the nuchal cord group compared to controls (128 ± 81 vs. 141 ± 80 min, respectively; *p* < 0.001; Table 2).

No difference was found in the rate of umbilical cord pH < 7.1 between the groups (54 cases (5.1%) in the nuchal cord group and 107 (4.0%) in the control group (*p* = 0.89)). There was no difference in Apgar scores or in the rate of NICU admissions between groups. The rates of 5 min Apgar scores ≤ 7 were 11 (1%) in the nuchal cord group and 25 (0.9%) in the control group (*p* = 0.751). Mechanical ventilation was required in 6 cases (0.6%) in the nuchal cord group and in 12 cases (0.45%) in the control group (*p* = 0.628). Phototherapy was administered to 77 patients (7.3%) in the study group and 192 (7.1%) in the control group (*p* = 0.875). NICU admission was reported in 18 cases (1.7%) in the nuchal cord group vs. 41 cases (1.5%) in the control group (*p* = 0.693; Table 3).

To assess whether multiple (≥2) nuchal cord is a risk factor for adverse outcomes, all the VAD with multiple nuchal cord were compared to VAD without nuchal cord. There were no differences between groups in the rate of low 5 min Apgar scores, lower cord pH, NICU admissions, subgaleal hematoma, shoulder dystocia, clavicular fracture or third- or fourth-degree perineal tear (Table 4).

Multivariate logistic regression was used to assess factors associated with nuchal cord. The logistic regression was adjusted for maternal BMI, labor induction, nulliparity, NRFHR as indication for VAD, fetal birthweight and second-stage duration. It was found that parous women, labor induction and lower fetal birthweight were independent risk factors for VAD with nuchal cord (Table 5).

## 4. Discussion

This study focused on singleton VAD that were diagnosed with nuchal cord postpartum. The main findings were lower rate of nulliparity, higher BMI and lower average fetal birthweight in the group of VAD with nuchal cord. The rate of induction of labor and NRFHR as indications for VAD were higher in the nuchal cord group. There was no difference in the rate of umbilical cord pH < 7.1 between the two VAD groups.

Like other studies [1,16], the average birthweight in the nuchal cord group was lower than in the control group. However, the rate of SGA newborns (according to gestational age) was similar. In this circumstance, it is not clear what is the cause and what is the result. Possibly, the blood supply of fetuses with nuchal cord is interrupted and therefore, they are smaller. However, it is possible that smaller fetuses tend to move more and thus, the rate of nuchal cord is higher in this group.

Whether nuchal cord is associated with higher frequency of labor induction or higher rate of NRFHR is debatable [10,11,17]. According to our data, a greater proportion of pregnant women with nuchal cord in whom VAD was performed underwent induction. In addition, there was a higher rate of VAD due to NRFHR in the nuchal cord group. However, the rate of umbilical cord acidemia (pH < 7.1) in the nuchal cord group was not higher.

Several studies showed that multiple nuchal cord is associated with adverse outcomes [18,19,20]. According to our data, VAD with ≥2 nuchal cords is not associated with additional risk for adverse outcomes compared to VAD without nuchal cord. A possible explanation is that a multiple nuchal cord increases the risk for VAD, which is the main factor for the increased rate of adverse outcomes. However, these results may be due to the relatively small sample size. Note, there was a tendency for higher rate of cord pH < 7.1 in the multiple nuchal cord group, but without statistical significance (14 cases (6.7%) vs. 107 cases (4.0%), *p* = 0.068).

The clinical significance of our findings is that since the presence of nuchal cord did not affect the outcome of VAD, there is no reason to look for nuchal cord or to report its presence during an ultrasound scan. These results are in accordance with previous studies which reported that nuchal cord is not associated with adverse outcomes [1,17].

The limitations of this study are related to its retrospective design, and that data regarding long-term outcomes are lacking. The strengths of the study include that data were collected from a single institution with a uniform protocol for VAD. In addition, the relatively large study population allowed us to control for various confounders.

## 5. Conclusions

From the perspective of VAD, our data support the approach that screening for nuchal cord or reporting its presence is not recommended, given the lack of evidence regarding adverse outcomes. Further studies are needed to assess the significance of nuchal cord with multiple loops in this situation.

## Figures and Tables

**Table 1:** Baseline characteristics of the study groups.

Variable	Nuchal Cord Group (*n* = 1059)	Control Group (*n* = 2695)	*p*-Value
Maternal age (years ± SD)	30.12 ± 5.4	37.8 ± 2.6	0.427
Gestational age (weeks + days ± SD)	39 + 3 ± 1.3	39 + 3 ± 1.4	0.685
Nullipara, *n* (%)	729 (68.8)	2004 (74.4)	0.001
Body mass index (kg/m^2^)	23.6 ± 4.3	23.1 ± 4.5	0.017
DM, *n* (%)	74 (7.0)	37 (10.6)	0.735
Smoking, *n* (%)	53 (5)	177 50.9)	0.884
Vaginal birth after cesarean, *n* (%)	77 (7.3)	208 (7.7)	0.640
Hypertension/Preeclampsia, *n* (%)	27 (2.5)	52 (1.9)	0.234

DM, includes diabetes mellitus and gestational DM. Data are presented as mean ± SD or *n* (%).

**Table 2:** Labor and delivery characteristics in relation to nuchal cord.

Variable	Nuchal Cord Group (*n* = 1059)	Control Group (*n* = 2695)	*p*-Value
Induction, *n* (%)	207 (19.5)	431 (16)	0.009
Meconium, *n* (%)	203 (18.5)	458 (17.0)	0.115
Epidural, *n* (%)	944 (89.1)	2695 (90.6)	0.93
2nd-stage duration (min ± SD)	128 ± 81	141 ± 80	<0.001
Head position-OA (*n*, %)	809 (76.4)	2095 (77.7)	0.617
Head station			0.617
S + 1 (*n*, %)	567 (55.6)	1502 (57.8)	
S + 2 (*n*, %)	422 (41.4)	1013 (39.0)	
S + 3 (*n*, %)	22 (2.2)	60 (2.3)	
Missing data	48 (4.5)	120 (4.4)	
Vacuum indication			0.004
NRFHR, *n* (%)	830 (80.7)	1989 (75.6)	
Prolonged 2nd stage, *n* (%)	186 (18.1)	607 (23.1)	
Other, *n* (%)	43 (4.1)	99 (3.7)	
Cup type			0.522
Kiwi, *n* (%)	690 (65.2)	1726 (64.0)	
Sylastic, *n* (%)	369 (34.8)	969 (36.0)	
Cup detachment, *n* (%)	212 (20.1)	566 (21.0)	0.514
True knot, *n* (%)	10 (0.9)	25 (0.9)	0.962
Episiotomy, *n* (%)	1573 (76.1)	250 (71.8)	0.087
Birthweight, (g ± SD)	3185 ± 413	3223 ± 436	0.013
Small for gestational age, *n* (%)	113 (10.7)	255 (9.6)	0.262

NRFHR, Non-reassuring fetal heart rate. Data are presented as mean ± SD or *n* (%).

**Table 3:** Maternal and neonatal outcomes stratified by nuchal cord group.

Variable	Nuchal Cord Group (*n* = 1059)	Control Group (*n* = 2695)	*p*-Value
5 min Apgar ≤ 7, *n* (%)	11 (1.0)	25 (0.9)	0.751
Cord pH ≤ 7.15, *n* (%)	103 (9.7)	249 (9.2)	0.890
Cord pH ≤ 7.1, *n* (%)	54 (5.1)	107 (4.0)	0.188
Neonatal intensive care unit admission, *n* (%)	18 (1.7)	41 (1.5)	0.693
Mechanical ventilation, *n* (%)	6 (0.6)	12 (0.45)	0.628
Phototherapy, *n* (%)	77 (7.3)	192 (7.1)	0.875
Subgaleal hematoma, *n* (%)	45 (4.2)	141 (5.2)	0.228
Shoulder dystocia, *n* (%)	17 (1.6)	42 (1.6)	0.917
Clavicular fracture, *n* (%)	8 (0.8)	22 (0.8)	0.850
Third/fourth-degree perineal tear, *n* (%)	20 (1.9)	60 (2.2)	0.519

Data are presented as mean ± SD or *n* (%).

**Table 4:** Maternal and neonatal outcomes stratified by multiple (≥2) nuchal cord.

Variable	Multiple Nuchal Cord (*n* = 208)	No Nuchal Cord (*n* = 2695)	*p*-Value
5 min Apgar ≤ 7, *n* (%)	2 (1)	25 (0.9)	0.999
pH ≤ 7.15, *n* (%)	27 (13.0)	249 (9.2)	0.106
pH ≤ 7.1, *n* (%)	14 (6.7)	107 (4.0)	0.068
Neonatal intensive care unit admission, *n* (%)	0 (0)	41 (1.5)	0.115
Subgaleal hematoma, *n* (%)	6 (2.9)	141 (5.2)	0.137
Shoulder dystocia, *n* (%)	1 (0.5)	42 (1.6)	0.366
Clavicular fracture, *n* (%)	1 (0.5)	22 (0.8)	0.999
Third/fourth-degree perineal tear, *n* (%)	4 (2.0)	60 (2.2)	0.699

**Table 5:** Multivariate analysis model for delivery characteristics in relation to VAD with nuchal cord.

Variable	*p*-Value	OR	95% Confidence Interval	
			Lower	Upper
Maternal BMI	0.087	1.02	0.997	1.044
Parous	<0.001	1.64	1.26	2.14
Labor induction	<0.001	0.66	0.53	0.81
Second-stage duration	0.713	1.00	1.00	1.00
NRFHR	0.396	1.14	0.85	1.53
Birthweight	0.002	1.00	0.99	1.00

Multivariate analysis model adjusted for maternal BMI, parous, labor induction, second-stage duration, NRFHR as indication for VAD and birthweight.

## Data Availability

Data will be made available from the corresponding author upon reasonable request.
